# Framing older adults’ loneliness in Danish news media: between societal responsibility and individual burden

**DOI:** 10.1093/geronb/gbag063

**Published:** 2026-04-09

**Authors:** Axel Ågren, Elisabet Cedersund, Christine Swane

**Affiliations:** Department of Culture and Society (IKOS), Division of Social Work, Linköping University, Linköping, Sweden; Department of Culture and Society (IKOS), Division of Ageing and Social Change, Linköping University, Linköping, Sweden; Department of Culture and Society (IKOS), Division of Ageing and Social Change, Linköping University, Linköping, Sweden; EGV Foundation—Social Inclusion of Older Adults (Fonden Ensomme Gamles Værn), Copenhagen, Denmark; (Social Sciences Section)

**Keywords:** Age norms, Active ageing, Compassionate ageism, Discourse analysis, Othering

## Abstract

**Objectives:**

Loneliness among older adults is addressed as a global public health issue and constitutes a key policy issue, internationally and within countries. Denmark is a country where numerous policies, initiatives, and campaigns, with aims of reducing loneliness, have been conducted over the past decade. The news media influence public understandings of loneliness and how the issue is dealt with in policy and practice. The aim of this study is to examine how loneliness among older adults is constructed in the Danish news media.

**Methods:**

Danish news articles were analyzed through the lens of Norman Fairclough’s critical discourse analysis, with emphasis on the interplay between media representations and societal discourses in the construction of loneliness.

**Results:**

Four discourses were identified in the analysis: (1) a discourse of social activities to reduce loneliness; (2) a discourse of loneliness as a societal and political issue; (3) a discourse of housing and the importance of the physical environment for reducing loneliness; and (4) a discourse of lived experiences of loneliness in later life.

**Discussion:**

Articles were dominated by a focus on activity-based interventions, in which politicians, project managers, volunteers, and older adults were given a voice. Structural conditions, shortcomings in eldercare provision, and portrayals of older adults as “forgotten” were constructed as primary causes of loneliness. In these articles, older adults were portrayed as victims and were not present. It is important to maintain a critical awareness of the role the news media plays in constructing images of ageing, old age, and loneliness.

In recent years, loneliness among older adults has attracted considerable international attention as a major global social problem and a crucial policy concern affecting societies, organizations, and individuals (World Health Organization, 2021; Yang, 2019). Research indicates that loneliness may negatively affect health across the life course. In fact, middle-aged and younger adults may be at a higher risk of experiencing and being affected by loneliness than older adults (Barreto et al., 2021; Holt-Lunstad et al., 2015). Public and media discourses nevertheless tend to construct older adults as particularly at risk of loneliness (Ågren & Cedersund, 2020; Jentoft et al., 2024). Existing studies point to stereotypical views of older adults as lonelier than other age groups (Dykstra, 2009; Tornstam, 2007).

Although loneliness is often considered a central aspect of human existence, its meanings and experiences are culturally dependent (Johnson & Mullins, 1987). The media plays a key role in constructing these meanings by reflecting cultural beliefs and myths, while simultaneously shaping public understandings of aging, later life, and older adults as a homogeneous group associated with problems such as loneliness (Ågren, 2017; Amundsen, 2022; Reul et al., 2022). Moreover, culturally embedded ageist beliefs may be internalized from an early age and reinforced across the life course (Levy et al., 2020). Such internalization may result in negative self-perceptions of aging, which in turn influence experiences of loneliness, health, and well being and lead to substantial healthcare costs (Levy et al., 2020; Pikhartova et al., 2016).

Denmark is an example of a country that has experienced extensive societal attention to loneliness among older adults across policies, media, and organizational arenas. In 2016 and 2017, numerous projects, reports, campaigns, and evaluations focusing on loneliness were initiated and implemented (see Christiansen & Lasgaard, 2017; SFI—Det Nationale Forskningscenter for Velfærd, 2017). These years were formative for the establishment and subsequent development of initiatives aimed at reducing loneliness among older adults in Denmark. Examples of such initiatives include national awareness campaigns seeking to reduce stigma surrounding loneliness, community-based meal programs, public dissemination about loneliness, and cooperations between state actors and civil society organizations (see United against Loneliness: https://modensomhed.dk/; DaneAge Association: https://www.aeldresagen.dk/; EGV Foundation: https://egv.dk/). This substantial attention raises the question of how the news media engage with the issue of loneliness and how different actors involved in efforts to reduce loneliness are represented and given a voice in news media coverage. Examining news media constructions of loneliness among older adults is important, as news coverage tends to reproduce stigmatizing and ageist views of older adults as frail, dependent, and lonely (Ågren, 2017). Furthermore, public discourses on loneliness are often dominated by normative assumptions that frame loneliness as a problem that needs to be fixed (Ågren, 2017; Sagan, 2024). Loneliness is, however, a complex phenomenon. A substantial body of research has documented its significant influence on older adults’ physical and mental health negatively, motivating the need for campaigns, projects, and interventions. At the same time, loneliness is a societal concern, where public and media discourses, often through the use of dramatizing phrases, may reinforce portrayals of loneliness in later life as inherently problematic, potentially contributing to further stigmatization.

Against this backdrop, this study examines how loneliness among older adults is constructed in the Danish news media during a period of heightened societal attention to the issue. In addition, given the involvement of multiple actors in efforts to reduce loneliness, the study explores the interplay between political actors, civil society organizations, welfare institutions, and older adults in news media coverage. Particular attention is paid to how these actors are given space to articulate their perspectives and how their contributions shape dominant understandings of loneliness in later life.

## Background

### The role of news media in constructing old age and loneliness

Research addressing loneliness among older adults in the news media remains limited, despite the extensive media attention to the issue. In an analysis of how loneliness among older adults is constructed in the news press, Ågren (2017) found that primary attention was directed towards political issues, resource allocation, and the organization of eldercare, while loneliness itself received comparatively limited focus (Ågren, 2017). Thus, loneliness functioned primarily as a device to strengthen arguments about current deficiencies within the welfare state and eldercare system, the need for increased resources, and political change. With regard to the designation of responsibility for reducing loneliness, Ågren and Cedersund (2020) found that mainly welfare state institutions were held responsible, while problems of older adults were articulated by “non-old” actors. Thus, attempts at inclusion simultaneously produced forms of exclusion (Ågren & Cedersund, 2020). Similarly, in a study of loneliness in Finnish newspapers and a lifestyle magazine, Uotila et al. (2011) demonstrated that loneliness was discussed from an “outsiders’ perspective.” Older adults were frequently represented in negative terms, with loneliness constructed as a consequence of aging, illness, dependency, and decline (Uotila et al., 2011).

### Theoretical perspectives on how news media construct understandings of loneliness

In analyzing news media articles, our perspective on loneliness among older adults is grounded in a constructionist understanding of the social world as not simply existing “out there” (Berger & Luckmann, 1966), but as constituted through meaning-making practices, including written language in the news media. News media plays a central role in shaping public understandings of welfare issues (Misra et al., 2003; Yoo, 2001), particularly those located at the intersection of private troubles and public concerns (Conrad, 1997). However, the news media are not a source of objective reports on “reality” (Thompson, 2000). Rather, media communicate symbols that are perceived as meaningful within specific societal contexts. According to Seale (2003), a key feature of mass media representations is the creation and exploitation of opposites with the aim of creating drama. Dramatized contradictions and simplifications are central to all narratives and necessary for the media to entertain their audiences (Seale, 2003). At the same time, the news media constitute a complex field in which journalists, state actors, civil society organizations, industry representatives, and other stakeholders are intertwined, resulting in multiple sources of knowledge being available while blurring the boundaries between “expert” and “lay” knowledge (Hallin et al., 2013). To understand the process of how various actors contribute to shaping public perceptions of loneliness in later life, theories of social problems are instructive. According to Loseke (2003), actors striving to draw public attention to potentially harmful conditions construct them as social problems by persuading audiences that they are widespread, harmful, and amenable to human intervention. These theories direct attention to whether loneliness is framed as an issue of individual responsibility or a structural condition, which is a central issue in studies on how social problems are represented in the news media (Oleschuk, 2020).

## Methodology

### Design

We draw on Fairclough’s understanding of discursive practices within the news media as constitutive of the social world, insofar as texts are produced and consumed within specific social contexts (Fairclough, 1995). From this perspective, media produce discourses – but at the same time, media discourses are themselves shaped by broader social structures, including political institutions and wider social developments beyond the media field. News media discourse is therefore understood as both shaped by and shaping social structures, contributing to their reproduction as well as their transformation (Fairclough, 1992).

### Empirical material

The empirical material consists of articles published in Danish national and regional newspapers over the years 2016, 2017, and 2025. The years 2016 and 2017 were selected as they marked a formative period during which multiple initiatives aimed at reducing loneliness were launched. Since loneliness has gained much attention, in Denmark and globally, over the past decade, we have included news articles from 2025 in our empirical material. This temporal selection enables a comparative analysis of whether and how media discourses on loneliness in later life have evolved over time, hence drawing on Fairclough’s concept of intertextuality, focusing on how meanings of texts build on each other and constitute and restructure conventions from prior texts (Fairclough, 1992). Articles were retrieved from Infomedia (https://www.infomedia.dk) using the Danish search terms “ensom gammel” (translated as “lonely older person”). Exclusion criteria comprised duplicate articles, press releases, brief notices, advertisements, and event announcements. Following the screening process, the final dataset consisted of 103 news articles (see Supplementary Table 1), which were downloaded and stored for analysis.

### Process of analysis

After identifying and retrieving the articles, the process of qualitative text analysis was initiated. In the initial phase of analysis, we read all the news articles several times to familiarize ourselves with the material. We subsequently proceeded with initial coding, which, according to Braun and Clarke (2006), entails identifying features of the data that are relevant to the research questions and can be meaningfully analyzed. This stage involves systematically organizing the dataset. Table 1 illustrates the coding process. Following the generation of initial codes, these were reviewed, refined, and grouped based on identified similarities and patterns. As Braun and Clarke (2006) note, tensions and contradictions are inevitable in extensive qualitative datasets. Acknowledging this view, we adopted a reflexive approach to the material, remaining attentive to similarities, differences, and variations across the dataset. However, some degree of categorization was necessary to facilitate analysis. In doing so, we sought to maintain transparency by explicitly addressing variations and complexities within the material.

**Table 1:** Examples of coding.

Excerpts (translated from Danish to English)	Codes
**The air was filled with kale at the Sofiegården Care Center on Tuesday at lunchtime, and there was a very special reason for that. The center had been visited by around 40 pensioners who were participating in the municipality’s latest initiative to prevent loneliness. The project has been named Old Regional Dishes, and consists of communal meals for the municipality’s elderly, who can thus meet new people (Vejle Amts Folkeblad. December 21, 2016).**	Eating together Preventing loneliness in later life The project in focus
**The Red Cross and Elderly Section in Store Heddinge department has, in collaboration with Stevns Volunteer Center and Stevns Municipality, started a new project called “Folgevenner.” “Folgevenner” is designed to ensure that elderly lonely citizens in Stevns Municipality can get help and support to get out into one of the many communities that exist (Stevns, 28 March 2025).**	Collaboration between NGO and municipality Help lonely older adults Become active

*Note*. NGO = Non-Governmental Organization.

Following the initial phase of reading, organizing, and coding the empirical material, we proceeded to a more in-depth analysis. In line with the study’s aim and overarching theoretical framework, the analysis focused on how loneliness among older adults was constructed in the Danish news media. We drew on elements of Fairclough’s (1992) three-dimensional model of critical discourse analysis, attending to (1) textual features, (2) processes of text production, and (3) the broader social practices within which communicative events occur. At the textual level, we examined vocabulary, recurring phrases, and sentence structures that provided insight into how discourses of loneliness were constituted. For example, loneliness was frequently articulated through references to specific activities aimed at reducing it, with shared meals emerging as a recurrently promoted solution. Drawing on Fairclough’s emphasis on genre and the ordering of discourse (1995), we also analyzed how the news media as a genre shaped representations of loneliness. A recurring pattern was the juxtaposition of depictions of loneliness as a serious and urgent problem with positively framed accounts of initiatives to reduce loneliness. In line with Fairclough’s emphasis on the process of text production and social practices where communication occurs, we examined which actors were represented. We found that politicians recurrently described loneliness as a major problem in later life and portrayed older adults as “forgotten” in contemporary society. In articles focusing on specific activities, volunteers were often foregrounded, with detailed attention paid to the organization of activities, the rationale for addressing loneliness, and on-site reportage of events. Experts were occasionally included to provide their perspectives on loneliness, drawing on findings from research and surveys.

When older adults were interviewed, they frequently described having previously experienced loneliness, while emphasizing that participation in organized activities had restored joy to their lives. Only a small number of articles focused exclusively on older adults’ own experiences of loneliness without introducing a “positive twist,” that is, without describing older adults’ strategies to alleviate their own loneliness.

The preceding account illustrates how elements of Fairclough’s theoretical and methodological framework informed our analytical process. The findings of the analysis are presented and discussed in the sections that follow. Throughout the process of analyzing the material and preparing this article, we adhered to the *Standards for Reporting Qualitative Research* (O’Brien et al., 2014), emphasizing transparency regarding data collection, analytic procedures, theoretical framing, methodological approach, findings, and conclusions.

## Results

In this part, we will present the discourses that emerged through the analysis of the empirical material (Table 2). See Table 3 for each excerpt referenced in this section.

**Table 2:** Summary of discourses identified.

Discourse	*N* articles
**Social activities to reduce loneliness**	53
**Loneliness as a societal and political issue**	45
**Housing and the importance of the physical environment for reducing loneliness**	3
**Lived experiences of loneliness in later life**	2

**Table 3:** Excerpts.

Excerpt #	Excerpts (translated from Danish to English)	Newspaper	Publication date
**1**	My network is no longer that large, so for me, every Thursday is a highlight. Coming down here to eat good food in a cozy company with old friends is just amazing. Enjoying the company of the “Dining Friends” helps to keep loneliness away. I couldn’t do without this …	Lokalavisen Egedal	December 27, 2016
**2**	You can sometimes feel trapped inside nursing homes, where there are few activities, and you are left to yourself. I have tried to get to know others through church because I am very lonely, but nothing came out of that. Bike rides give me so much joy in life and a feeling of freedom and autonomy, even if I do not ride the bike myself.	Kristeligt Dagblad	June 1, 2017
**3**	The Red Cross and Ældre Sagen in Store Heddinge have, in collaboration with Stevns Volunteer Center and Stevns Municipality, started a new project called “Følgevenner”. “Følgevenner” is designed to ensure that elderly lonely citizens in Stevns Municipality can get help and support to get to one of the many communities that exist in Stevns Municipality […]—There are quite a few citizens in Stevns Municipality who are alone at home or have lost a spouse or friends as they get older, or they may be new to Stevns Municipality and thus not familiar with the community life in Stevns.	Stevns	March 28, 2025
**4**	There are a lot of lonely people out there. I have a friend who said to me directly: “I’m lonely.” And then he just looked at me.	Helsingør Dagblad	September 20, 2025
**5**	We know that Sunday is often a long day for many. Sunday’s empty silence does not always feel comfortable for all. It’s a bit like Christmas, which can also be hard to get through […]. Loneliness is perhaps the greatest plague of our time, and one of the most devastating. But there are very few lonely people who will stand up and say [that they are lonely], even if no one comes to visit….	Dagbladet Holstebro	January 19, 2017
**6**	When you live in one of the municipality’s nursing homes, the days can quickly become monotonous and characterized by isolation within the four walls of the center. It is a situation that we, as a society, have a responsibility to take seriously.	Randers Amtsavis	October 4, 2025
**7**	Having been a widow for 13 years, she has grown used to being alone, as much as you can after 48 years of marriage. It took a few years. But what she will never get used to is the void after her son who was taken away from her due to cancer. She does not experience this as a lack of sounds around her or an absence of company, but as a heavy feeling of grief that lives inside her, a grief which is a pervasive element in her story about her loneliness. […] When she feels the loneliest, she cries. She cries over how she only hears the sounds she makes in the small, terraced house, over how she hasn’t spoken to anyone for days, and how she feels that she has no one with whom to share the great sadness of her life, but also the little joys of everyday life.	Jyllands-Posten	July 13, 2017

### Discourse of social activities to reduce loneliness

Across the material, loneliness was frequently constructed as a problem that can be alleviated through various organized social activities. In an article about a nationwide initiative, “Dining Friends,” an older woman’s perspective on the activity at hand was included (*Excerpt 1*).

The quote illustrates how an older woman talks about the companionship facilitated by participation in the “Dining Friends” initiative. In the same article, the project manager describes the initiative, arguing that the older adults she met became like family to her and how it gave lonely older adults and people with dementia “…a feeling of safety and community that is indescribable” (Lokalavisen Egedal, December 27, 2016). This article illustrates how activities to reduce loneliness are constructed by describing the activity in detail, emphasizing the importance of loneliness alleviating initiatives, and suggesting how to enable older adults to participate in social activities. Across the articles, eating together emerged as a recurrently promoted solution to loneliness by enabling togetherness. However, this predominantly positive framing was not uncontested. A local newspaper ran an article under the headline of “Older adults do not want to eat together,” reporting limited interest in a municipality’s investment in “Dining Friends” with the invitation to eat together with a volunteer (Vejle Amts Folkeblad, December 21, 2016). In the article, the chairman of the municipality’s senior committee was interviewed about efforts to enable older adults to eat together with a volunteer. Over a 2-year period, only two people had made use of the service. The chairperson questioned whether there were in fact that many lonely people or whether older adults simply did not want such forms of organized activity. Notably, on the same newspaper page, another article described a different shared-meal initiative under the headline: “New project against loneliness had a promising start.” In this case, home care recipients were invited by care staff to dine at a nursing home. The article gave voice to participating older adults as well as managers and politicians, and it emphasized sensory and affective dimensions; for instance, describing how “a faint scent of hamburger with kale hung in the air” (Vejle Amts Folkeblad, December 21, 2016).

Although shared meals dominated the coverage, other activities were also presented as remedies for loneliness. One example involved volunteers offering rickshaw bicycle rides to nursing home residents. The initiative was framed as enhancing quality of life, restoring dignity, and enabling nursing home residents to reconnect with society. The article foregrounded volunteers’ efforts, motivations, and personal lives. Two researchers were cited to describe the positive impact that cycling may have on older adults. The article does, however, also describe the activity from the perspective of older adults, as illustrated in *Excerpt 2.*

The article foregrounds activity, getting out into nature, and the pursuit of companionship. At the same time, the excerpt illustrates how an older woman justifies her participation in the rickshaw initiative by describing herself as “very lonely,” feeling “trapped” in a nursing home, and unable to find activities in church. In this way, the excerpt conveys a critique of institutional life in nursing homes. Unlike the generally positive framing of social activities, this account suggests that social participation does not automatically reduce feelings of loneliness. Although the excerpt does not explicitly state whether her feelings of being “very lonely” are reduced, participating in the project resulted in “so much joy,” freedom, and autonomy.

In 2025, the discourse of social activities appeared less prominent compared to 2016 and 2017. Nevertheless, news articles continued to highlight issues of initiatives aimed at reducing loneliness, as illustrated in *Excerpt 3.*

Similar to articles published in 2016 and 2017, the activity itself occupied the larger part of the article, with primary attention directed toward actors conducting the activity and how it was structured and organized.

The type of activity reported on, as well as the way it was framed, appeared to influence how loneliness was constructed. As noted above, shared meals were a recurrent theme in articles from 2016 to 2017. These articles were overall framed in positive terms, and older adults were given relatively extensive space to articulate their own motives for participating and their personal experiences of loneliness. By contrast, in the articles from 2025, loneliness was more frequently addressed from an “outsiders’ perspective,” with the voice of organizers and facilitators foregrounded, as illustrated in *Excerpt 4.*

The notion that many older adults are sitting in loneliness in society was recurrent in the 2025 articles. Notably, such claims were rarely substantiated by references to research, survey data, or professional expertise. Instead, they were often framed as expressions of personal belief or assumption, as illustrated in the following statement: “We have a feeling that there are many lonely elderly people living in this area…” (Rudersdal, September 9, 2025).

### Discourse of loneliness as a societal and political issue

Loneliness was constructed as a societal and political issue in several articles. Although articles in this discourse revolved around specific activities, their primary focus was to address loneliness as a problem caused by societal structures.

One article, for example, described the idea behind a “Sunday coffee” initiative launched by deacons in a local Christian parish. The initiative relied on volunteers inviting individuals into their homes for a chat, coffee, and cake. In the article, a clergyman involved in the initiative was quoted as stating: See *Excerpt 5*.

*Excerpt 5* illustrates several dimensions of how loneliness is framed within this discourse. It draws on normative expectations that holidays and festive occasions ought to be spent in the company of others, positioning solitude as particularly problematic. Loneliness is explicitly constructed as a societal concern, as it is “the greatest plague of our time,” while the interviewee also points to a broader unwillingness to reveal loneliness in contemporary society.

Under the headline “Remember the old–all year round,” another article opens by portraying Christmas as a holiday during which older adults are expected to gather with their grandchildren around a well-laid table. According to the former Danish minister for senior citizens, the reality is that primarily older adults spend Christmas alone, which can make this holiday “especially challenging.” The former minister argued that although the public sector and civil society contribute substantially, including investments in future homecare, everyone in the family also carries responsibility to invite and include older family members throughout the year, despite a busy everyday life, if “… we as a population really want to do something to end loneliness among older adults” (Nordjyske Stiftstidende, December 26, 2016). The article reveals discursive duality. On the one hand, “we” are implicitly positioned as contributing to loneliness insofar as older adults are forgotten by “society.” On the other hand, the same collective “we” is constructed as the solution: responsible for remembering and including older adults year-round in order to reduce loneliness among “them.” A similar association between Christmas, loneliness, and old age appeared in a local newspaper, where Christmas was described as the time of year when relations with older relatives are temporarily “polished,” while they are forgotten throughout the rest of the year. The author further argued that Christmas can be particularly demanding for frail and lonely older adults with hearing and visual impairments, who may prefer to meet their family on less hectic days than Christmas Eve. The author concluded that “we” should make efforts to care for “our elderly,” through more frequent phone calls and taking them for a drive (Frederiksborg Amts Avis, December 16, 2025).

The understanding of Christmas as an important holiday during which older adults should not be left alone or forgotten illustrates how loneliness is discursively linked to societal norms. Christmas functions as a symbolic amplifier, intensifying the argument that older adults are forgotten. Furthermore, the assertion that older adults should not be forgotten, during Christmas or throughout the year, can be understood through the concept of compassionate ageism (Binstock, 2010), where older adults are constructed as victims who are entitled to help from “society.”

The party leader of the Danish Social Democrats at the time argued in a local newspaper (Fyens dk, November 7, 2017) that loneliness is rooted in broader societal developments. Specifically, she linked loneliness to cutbacks in the care sector, resulting in “fewer hands” in eldercare and a reduction in activities for older adults. In this framing, loneliness is constructed not as an individual issue, but as a structural consequence of political and economic priorities. Additionally, changing family patterns were described as contributing to loneliness. Younger generations were described as lacking time to meet older relatives, while older adults were depicted as unable to structure their days around social activities. These developments were framed as the downside of modern society. These findings are echoed in other studies, in which developments in modern society are viewed as exacerbating loneliness among older adults (Ågren & Cedersund, 2020; Jentoft, 2023).

Several articles within this discourse addressed the situation concerning eldercare. One article opened with a general portrayal of home care today, arguing that staff move quickly in and out of people’s homes and that the days when staff would cook together with older adults or have time to sit down and hold the hand of a sick or bedridden person belong to the past. A researcher interviewed in the article argued that the growing proportion of older adults, combined with fewer “warm hands” in care, raises the question of whether there will ever be time simply to share a cup of coffee. According to the researcher, this development points to the need for increased involvement of civil society, suggesting that family members, neighbors, and others will have to assume a greater role in the care of older adults. Similarly, a director of a center for political studies described contemporary care work as subject to a “tyranny of the clock” and how care for older adults involves a great deal of time-consuming documentation, leaving staff feeling inadequate (Kristeligt Dagblad, November 17, 2017). This issue was also addressed in two articles published on the same day in a local newspaper (JydskeVestkysten, February 24, 2016). Two of these articles were based on survey results indicating that care staff could do nothing to reduce loneliness as they had too little time for “meaningful conversations” or “small talk and a cup of coffee,” as their work had become increasingly governed by time constraints. Another article on the issue of problems within eldercare argued that voluntary befrienders have the time for lonely older adults that social and health professionals lack (JydskeVestkysten, February 24, 2016). In several articles, lack of time is constructed as a major societal problem. Care work is depicted as dominated by the measurement of minutes, hygiene and cleaning tasks, leaving insufficient time for conversation with older care receivers.

A debate-article written by a schoolteacher describes how he and a colleague found an old man lying in reeds by a lake. Together with staff from a nursing home, they pulled him out, after which he was able to walk on his own. The man said that he no longer wished to live and that no one visited him. According to the author of the article, “we all” bear responsibility “when lonely old people are sitting all around the country.” He asserts that “we” are not taking proper care of “our elderly” if circumstances have deteriorated to the point where they try to end their lives due to loneliness and isolation (Jyllands-Posten, February 14, 2017). The article then continues to state that more resources should be devoted to care staff, comparing it to other municipal expenditures. It is thus both up to “all of us” and to political decision-makers in the municipality to act to ensure that citizens do not spend their last years alone.

A similar construction appeared in Kristeligt Dagblad, where a former head of health and social care described the efforts made in Copenhagen to combat loneliness among older adults. The author argued that if we really want to end loneliness, everyone must think of how they themselves can contribute. Examples of suggested actions listed are inviting one’s grandma for coffee, taking the neighbor’s children to the playground, or simply talking to the neighbor. The author asserted that one’s contribution can make a big difference for those who want to be part of the community. She ended the article with the appeal: “Because if we are going to combat loneliness, it is important that both you and I lend a hand” (Kristeligt Dagblad, April 24, 2017).

Throughout the articles from 2025, resemblances were indeed found within this discourse, where it in fact emerged as the most dominant discourse of that year. Several articles centered on what was described as a crisis of eldercare, where cutbacks were seen as exacerbating loneliness, as illustrated in *Excerpt 6.*

In this article, the lack of time and resources is constructed as producing a monotonous everyday life for those older adults in nursing homes, particularly those without relatives. As in several other articles, the notion of “we as a society” is also invoked in this article. Moreover, the article forms part of a political debate, as the author represents an opposition party and claims that the governing party rejected a proposal for a so-called stamp card (allowing 30 min of conversation or help to get outdoors) to enhance the quality of life for older adults. A suggestion which “…gives lonely older adults a small but crucial boost in their everyday lives” (Randers Amtsavis, October 4, 2025).

### Discourse of housing and the importance of the physical environment for reducing loneliness

Three articles in the material highlighted, in different ways, the significance of housing and the physical environment in relation to reducing loneliness. Within this discourse, loneliness is constructed as spatially conditioned and potentially mitigated through living arrangements. One article presented an inherent tension in its headline: “Loneliness kills: Still old people prefer to live alone*”* (Ekstra Bladet, September 18, 2016). Researchers interviewed in the article suggested several explanations for older adults’ reluctance to live in collective housing: living alone may feel safer; they may not wish to adapt to others; they may have health problems; and financial or emotional issues may affect their preferences. In another article about collective housing, a representative of a community housing firm argued that residents live longer and experience less loneliness in such environments. Similarly, an article opening with the statement, “As you get older, you can quickly become lonely” (Fyns Amts Avis, April 18, 2017), featured interviews with residents about their reasons for moving into collective housing and why they enjoyed it. A sense of safety was frequently mentioned, for example, the assurance of having others nearby in case of illness. Across these articles, loneliness was repeatedly described as a motive for older adults to move to collective housing. When older adults were interviewed, however, loneliness was not articulated as a reason for having moved (Fyns Amts Avis, April 18, 2017).

### Discourse of lived experiences of loneliness in later life

Two articles stood out from the rest of the material in that they focused exclusively on describing the lived experiences of loneliness from the perspective of two women. Unlike most articles, which framed loneliness through social initiatives, policy debates, or housing solutions, these texts centered on personal narratives.

In a portrait of an 80-year-old woman, she reflected on her life course and current situation. The article emphasized the adversities she had faced: the early death of her husband, a hip fracture some years ago, and now the onset of dementia. Yet despite these challenges, she was portrayed as positive, active, and socially engaged. She described herself as always having been extroverted with a large social network. According to her, one cannot connect with others if one remains at home complaining about feeling sad and tired of being alone. “If you just sit at home, you come to a complete standstill,” she stated. She emphasized that she did not feel lonely as she kept in touch with friends, children, and grandchildren and often invited others for coffee (Dagbladet Holstebro, January 19, 2017). Another article had a similar focus, in which a personal portrait was made of an 84-year-old woman who described herself as very lonely. In this article, loneliness was closely related to loss, grief, and prolonged periods of solitude. Unlike the first portrait, this article did not emphasize maintaining an active lifestyle or presenting solutions to loneliness. Instead, it provided detailed examples of how profound the impact of feelings of loneliness is in her everyday life, as illustrated in *Excerpt 7.*

What emerged here is an in-depth portrayal of this woman, called “Grethe,” focusing on her experiences of loss and grief over the years and how these are intertwined with her sense of loneliness. Being alone is also distinguished from feeling lonely, as she has “gotten used to being alone,” yet still has persistent feelings of loneliness. In this way, the article uncovered the complexities of loneliness: later in the article, it was the fact of not sharing everyday life with another person that led her to cry from loneliness. In most of the articles analyzed, the negative aspects of loneliness were often overshadowed by a “positive twist” where a solution to loneliness was presented: sharing a delicious meal, allocating more resources to combat loneliness, a “we” striving to include older adults in society, or portraying older adults as active, capable of finding their own ways to overcome loneliness. In this particular article, however, loneliness is presented solely as a painful and enduring aspect of life for “Grethe,” with no solutions.

## Discussion

The aim of this study has been to analyze how loneliness among older adults is constructed in the Danish news media, with particular attention to the interplay between politics, civil society, welfare institutions, and individuals, as well as to who is given a voice in these representations. Our analysis shows that most news articles emphasize solutions aimed at reducing loneliness and highlight how activities enable fellowship and contribute positively to older adults’ lives. A wide range of actors, such as volunteers, politicians, researchers, care managers, and project leaders, appeared in the articles. Notably, however, older adults are also, at least to some degree, given the opportunity to describe their own lives, experiences of loneliness, and motivations for participating in activities. In this sense, the boundary between “expert” and “lay” knowledge appears blurred (Hallin et al., 2013). Different forms of knowledge are intertwined, resulting in diverse constructions of loneliness. Despite this plurality, loneliness among older adults is consistently framed as an urgent societal problem that requires intervention.

This finding is not in line with previous research suggesting that media articles about older adults are written from an “outsiders” perspective’ (Ågren & Cedersund, 2020; Nilsson, 2008; Uotila et al., 2011). What also emerged is a strong emphasis on the benefits of an active lifestyle, which has been discussed through the influential policy concept of active aging (Pfaller & Schweda, 2019). Ideals of activity are used as an important point of reference when older adults describe their quality of life, associating passivity with loneliness and various negative facets of aging (Ågren & Pavlidis, 2023; Kiuru & Valokivi, 2022). These ideologies have, in turn, been criticized for placing great responsibility on older adults who, due to structural inequalities, age stereotypes, diseases, or other life events, may be unable to meet such expectations (Calasanti & King, 2021). Parallel to studies on images of aging and older adults (Reul et al., 2022), we found that loneliness in the present material was situated within a tension between constructions of old age as either passive or active. In some articles, the primary focus was on activities, where interviews with professionals, volunteers, project managers, and older adults all contributed to construct understandings of the importance of maintaining a socially active lifestyle in later life. The activities were often given positive connotations through vivid descriptions of participants, delicious food, and music being played. Meanwhile, the darker sides of aging in contemporary society are associated with loss of autonomy, frailty, death, and loneliness (Higgs & Gilleard, 2014; Reul et al., 2022). These themes emerged in articles particularly addressing a perceived “crisis in care” and in the portrait of an older woman who presented her loneliness as associated with grief, loss, isolation, and disease.

Many articles, particularly within the discourse framing loneliness as a societal and political issue, highlight how society ignores lonely older adults. When comparing the news coverage from 2016 to 2017 with that from 2025, a notable shift becomes apparent. In the earlier period, initiatives aimed at reducing loneliness were the primary focus. These articles tended to portray older adults as active participants in society who engaged in initiatives and, at least implicitly, contributed to alleviating their own loneliness.

In contrast, the articles from 2025 were dominated by political and societal perspectives. Loneliness was increasingly framed as a consequence of welfare cutbacks and collective neglect. Responsibility was attributed to society, political decision-makers, and “all of us,” while older adults themselves were less frequently given a voice.

Here, old age was distinctly associated with loneliness, and the notion that older adults are forgotten was strongly foregrounded. Notably, older adults were not presented with names, photographs, or personal descriptions, nor were they interviewed. Instead, concerned citizens, care staff, and journalists articulated and discussed problems of care and how these lead to increased loneliness. It is, thus, mainly the problems with eldercare, lack of resources, insufficient time, and too few “warm hands” that were discussed, rather than loneliness from the perspectives of older adults themselves. This “outsiders” perspective’ resonates with what Loseke (2003) labels the *social problems industry*, in which actors who are not themselves directly affected by a problem construct certain conditions as potentially harmful. Moreover, these constructions resemble key arguments within the concept of “compassionate ageism” (Binstock, 2010), whereby expressions of concern for older adults are grounded in stereotypical images of frailty and dependency. Such portrayals risk positioning older adults as passive recipients of care rather than as active subjects (Jönson et al., 2023). This othering was further reinforced through phrases of how “we all” in society can and should act to end loneliness among older adults. The construction of a collective “we” simultaneously positions older adults as “them,” as a distinct and vulnerable group in need of intervention. An additional dimension emerged, namely the portrayal of “us” not only as responsible helpers but also as potential villains (Seale, 2003). Everyday social behaviors, such as avoiding sitting next to others on the bus, forgetting “our elderly,” or failing to invite people into our homes, were framed as contributing to loneliness. Hence, the question of who is responsible for causing—and resolving—loneliness becomes complex. On the one hand, abstract notions and critiques of the social fabric of everyday life advance structural perspectives, suggesting that “all of us,” and society as a whole, contribute to increasing loneliness in later life. On the other hand, older adults are, albeit often implicitly, constructed as responsible for “escaping” loneliness - “rewarded” with positive portrayal in news articles when they do so. Older adults who participate in activities to reduce their loneliness, such as sharing meals through “Dining Friends” and who thereby fulfill expectations of maintaining an active lifestyle, can be described as “lay” or everyday heroes. This framing reflects the growing prominence of confessional narratives and increased championing of the ordinary man and woman in the media (Seale, 2003). As Seale argues, the “lay hero” thesis has gained traction today in a society where professional authority has declined. The lay hero challenges orthodox approaches to, in this case, reducing loneliness. Hence, volunteers and the active older adult are positioned as role models whom we all can strive towards. Meanwhile, this also illustrates increased individualization, where the responsibility for escaping loneliness and mitigating the loneliness of others is increasingly assigned to the individual.

In line with prior studies on media and policy constructs of loneliness (Ågren & Cedersund, 2020; Jentoft, 2023), we nevertheless found that some news articles applied structural perspectives, attributing loneliness to broader developments of modern society, which resembles what Seale (2003) calls the “dangers of modern life.” These “dangers” were articulated in descriptions of contemporary society as characterized by increased individualization, taboos around loneliness, and shortcomings in care–and by the statement that “we” must learn to be more socially attentive in general and to more fully include older adults in our everyday lives.

## Implications

In sum, although the news media serve as an important source of information on loneliness in later life, they also contribute to constructing loneliness among older adults in particular ways. These constructions in turn affect policies, resource allocations (Yoo, 2001), and the design of interventions and methods for reducing loneliness. Future initiatives should therefore maintain a critical awareness of how news media constructions may reinforce stereotypical associations between old age and problems. Furthermore, although it is a difficult issue, increasing the participation of older adults and various other age groups in news media coverage of loneliness would increase our understanding and highlight that loneliness is not a phenomenon affecting only older adults.

## Supplementary Material

gbag063_Supplementary_Data

## Data Availability

This study was not preregistered. The data analyzed in this study consists of media articles and are thus publicly available. Contact the corresponding authors for inquiries.
