# Supplementary material for: Framing older adults’ loneliness in Danish news media: between societal responsibility and individual burden
Source: J Gerontol B Psychol Sci Soc Sci. 2026 Apr 9;81(6):gbag063. doi: 10.1093/geronb/gbag063 (PMC13180640; doi:10.1093/geronb/gbag063)
Supplement: gbag063_Supplementary_Data [file gbag063_supplementary_data.zip › JGSS suppl Ågren & Cedersund.pdf]

The Journals of Gerontology, Series B: Psychological Sciences and Social Sciences
Supplementary Material: Ågren & Cedersund. Framing older adults' loneliness in Danish news media: Between societal responsibility and individual burden.

Supplementary Table 1. Articles in the discourse of social activities to reduce loneliness by discourse type.

Article Title (Original in Danish)	English Translation of Article Title	Newspaper	Publication Date
**Discourse of social activities to reduce loneliness**			
1. Vågetjenesten er en værdifuld gave	The vigil service is a valuable gift	Magasinet Pleje	22 January 2016
2. Læsegrupper mod ensomhed	Reading groups against loneliness	Folkekirken dk	26 January 2016
3. Ensomhed bekæmpes med et godt måltid	Loneliness is combated with a good meal	Nordvestnyt	4 February 2016
4. Besøgsvenner erstatter plejepersonale	Visiting friends replace caregivers	JydskeVestkysten	24 February 2016
5. Til kamp mod ensomhed	Up To Fight Loneliness	Sjællandske Nyheder	16 March 2016
6. Den sociale muskel trænes i fællesskab	The social muscle is exercised together with others	Fyens Stiftstidende	26 April 2016
7. Odense spiser for et fælles mål	Odense eats for a common goal	Fyens Stiftstidende	26 April 2016
8. Lørdagsbrunch for to generationer	Saturday brunch for two generations	Fyens Stiftstidende	1 May 2016
9. Fonden med det provokerende navn	The Foundation with the Provocative Name	Magasinet Pleje	5 May 2016
10. København tager tungt våben i brug mod ensomhed blandt ældre: Babyer	Copenhagen is using a heavy weapon against loneliness among the elderly: Babies	Dagens dk	1 September 2016
11. Slut med ensomme ældre: Succesfuldt seniornetværk udbredes	No more lonely seniors: Successful senior network spreads	DR dk	8 October 2016
12. "Jeg føler mig ikke ensom, når jeg er her"	"I don't feel lonely when I'm here"	Egedal lokalavis	27 December 2016
13. Ældre ensomme skal til debat	Older lonely people need to be debated	Ballerup bladet	29 November 2016
14. Nyt projekt mod ensomhed kom lovende fra start	New project against loneliness got off to a promising start	Vejle Amts Folkeblad	21 December 2016
15. Ensomme ældre vil ikke have spisevenner	Lonely seniors don't want eating buddies	Vejle Amts Folkeblad	21 December 2016
16. Musikken trækker i ældre	Music attracts older people	Vejgaard Avis	21 December 2016
17. Herlevs hjemmepleje hjælper ensomme juleaften	Herlev's home care helps lonely people on Christmas Eve	Magasinet Pleje	23 December 2016
18. Stadig stor akut mangel på besøgsvenner	Still a great acute shortage of visiting friends	JydskeVestkysten	5 January 2017
19. Spisevenner måske på vej til Slagelses ensomme ældre	Dining friends perhaps on their way to Slagelse's lonely older adults	Sjællandske Nyheder	7 January 2017
20. Lykken er at have en besøgsven	Happiness is having a visiting friend	Hals Avis	10 January 2017
21. Ensomme ældre skal have hjælp af en app	Lonely older adults need help from an app	Dagbladet Roskilde	17 January 2017

Article Title (Original in Danish)	English Translation of Article Title	Newspaper	Publication Date
22. Omsorgen for hinanden - og hyggen sammen	Caring for each other - and having fun together	Horsens Posten	18 January 2017
23. Ensomme ældre i Aalborg Kommune skal hjælpes til stærkere sociale relationer	Lonely older adults in Aalborg Municipality must be helped to have stronger social relationships	Vodskov Avis	24 January 2017
24. En vej ud af ensomheden	A way out of loneliness	Midt-Vest Avis	25 January 2017
25. Projekt mod ensomhed hos ældre	Project against loneliness among older adults	Ugeavisen Svendborg	28 January 2017
26. Millioner til projekt om ensomhed	Millions for project on loneliness	Fyns Amts Avis	30 January 2017
27. Ensomhed hos ældre borgere skal bekæmpes	Loneliness among older adults must be combated	Odense dk	16 March 2017
28. Nyt netværk skal hjælpe ældre ud af ensomhed	New network aims to help older adults out of loneliness	Amtsavisen dk	10 April 2017
29. ”Oldekoller” er populære	"Eldercamps" are popular	Fokus	24 April 2017
30. Danmark spiser sammen i denne uge. Ensomhed skal bekæmpes af dig og mig	Denmark eats together this week. Loneliness must be fought by you and me	Kristeligt Dagblad	24 April 2017
31. Plejehjem i Aarhus får nye beboere - skaber stor glæde for de ældre	Nursing home in Aarhus gets new residents - creating great joy older adults	Dagens dk	15 May 2017
32. Ensomme ældre genfinder livsmodet i naturen	Lonely older adults rediscover the courage to live in nature	Friluftsrådet dk	17 May 2017
33. Seniorer cykler mod Malmø og mere livskvalitet	Seniors cycle towards Malmö and a better quality of life	Kristeligt Dagblad	1 June 2017
34. Workshop skal hjælpe ensomme ældre	Workshop to help lonely older adults	Amtsavisen	25 September 2017
35. Kirsten foretager dagens vigtigste opkald: Hun ringer til ældre og ensomme	Kirsten makes the most important call of the day: She calls the old and lonely	DR dk	12 October 2017
36. Jeg var ked af bare at kukkelure i min lejlighed	I was bored of just sitting around in my apartment	Jyllands-Posten	20 October 2017
37. En indsats for skæbner på kanten	An effort for those on the outside	Jyllands-Posten	5 November 2017
38. Biblioteker kan afhjælpe ensomhed hos ældre	Libraries can alleviate loneliness among older adults	JydskeVestkysten	28 November 2017
39. Biblioteker skal bryde ældres ensomhed	Libraries must break loneliness among older adults	Politiken	28 November 2017
40. Ensom enkemand satte seddel op i supermarked: Nu vælter det ind med jule-invitationer	Lonely widower put up a note in a supermarket: Now Christmas invitations are pouring in	Ekstra Bladet	1 December 2017
41. Opvask og julemad kan hjælpe mange ud af ensomhed	Washing dishes and Christmas food can help many people overcome loneliness	DR dk	24 December 2017
42. Ældres livskvalitet skal fremmes - det er ønsket bag donation	The quality of life of older adults must be promoted - that is the desire behind donation	Slagelse	22 January 2025
43. Ny hjælp til ensomme ældre	New help for lonely older adults	Stevns	28 March 2025

Article Title (Original in Danish)	English Translation of Article Title	Newspaper	Publication Date
44. Her behøver de ældre ikke frygte ensomhed	Here, older adults need not fear loneliness	Nordjyske	28 April 2025
45. Annette møder mindretallets ensomme ældre	Annette meets the lonely older adults of the minority	Flensborg Avis	5 May 2025
46. Ældre Sagen i Herning bakker op om senior-dating	Ældre Sagen in Herning supports senior dating	Herning Folkeblad	25 July 2025
47. »Gæsterne tager ikke med for at få en kæreste, men fordi de savner selskab«	"Guests don't come to get a girlfriend, but because they miss company"	Jyllands-Posten	12 August 2025
48. Nu skal naboer komme hinanden ved: - Det er nok det mest private sted, du kan ramme på kloden	Now neighbors have to get to know each other: - This is probably the most private place you can hit on the planet	Rudersdal	9 September 2025
49. 86-årige Niels ruller ud med varme og vilja	86-year-old Niels rolls out with warmth and will	Helsingør Dagblad	20 September 2025
50. SSF jubler: Nye kongeportrætter bevilget	SSF rejoices: New royal portraits granted	Flensborg Avis	17 December 2025
51. Godt nyt til svækkede ensomme ældre	Good news for frail lonely older adults	Horsens Folkeblad	18 December 2025
52. Røde Kors: Tak for hjælpen i 2025	Red Cross: Thank you for your help in 2025	Fyens Stiftstidende	27 December 2025
53. Middelfart Rickshaw Team siger tak efter rekordår	Middelfart Rickshaw Team says goodbye after record year	Fyens Stiftstidende	29 December 2025
**Discourse of loneliness as a societal and political issue**			
1. Ensomhed kan give dårligt helbred	Loneliness can cause poor health	Kristeligt Dagblad	19 February 2016
2. Sosu'er må efterlade triste ældre	Social and healthcare workers must leave behind sad elderly people	JydskeVestkysten	24 February 2016
3. Sosu'er kan intet gøre for ensomme ældre	Social and healthcare workers can do nothing for lonely older adults	JydskeVestkysten	24 February 2016
4. Ensomhed i en netværkstid	Loneliness in a networked age	Vejle Amts Avis	17 March 2016
5. Migræne, diabetes og hjertekarsygdomme er hyppigere blandt ensomme ældre	Migraines, diabetes and cardiovascular diseases are more common among lonely older adults	Defactum dk	1 April 2016
6. Måltidet er en social mulighed	The meal is a social opportunity	Fyens dk	1 May 2016
7. Undersøgelse: 45.000 ældre føler sig ensomme	Study: 45,000 seniors feel lonely	Kristeligt Dagblad	27 May 2016
8. Ensomhed i alderdommen er en diskussion med flere facetter	Loneliness in old age is a multifaceted discussion	Kristeligt Dagblad	10 July 2016
9. Husk de ældre - hele året	Remember the elderly - all year round	Nordjyske Stiftstidende	26 December 2016
10. Der er håb for ensomme	There is hope for the lonely	Jyllands-Posten	8 February 2017
11. Hvordan er det lige, vi passer på hinanden og vores ældre?	Do we really look after each other and our elderly?	Jyllands-Posten	14 February 2017
12. Ældres ensomhed brydes af et aktivt liv	Loneliness in later life reduced by activities	ElboBladet	12 April 2017
13. Sådan hjælper du den ældre ud af ensomheden	This is how you help an older person out of loneliness	Jyllands-Posten	2 May 2017

Article Title (Original in Danish)	English Translation of Article Title	Newspaper	Publication Date
14. S: Pulje på 100 mio. kr. til at bekæmpe ensomhed blandt ældre	S: Fund of 100 million DKK to combat loneliness among older adults	Fyens dk	7 November 2017
15. Er hjemmeplejen blevet så strømlinet, at der ikke er tid til menneskelig omsorg?	Has home care become so streamlined that there is no time for human care?	Kristeligt Dagblad	17 November 2017
16. Denne kvinde holder jul for ensomme: Man skal ikke sidde alene	This woman celebrates Christmas for the lonely: You shouldn't sit alone	TV2 Østjylland	29 November 2017
17. Ny landsby ved Thisted får hovedrolle i et forskningsprojekt med mål om at minimere ensomhed og fremme fysisk og psykisk sundhed blandt ældre og syge	New village near Thisted gets lead role in research project with goal of minimizing loneliness and promoting physical and mental health among old and sick people	TV Midvest dk	29 November 2017
18. Julen er både hjerternes og ensomhedens fest	Christmas is a celebration of both love and loneliness	Kristeligt Dagblad	15 December 2017
19. Kommunen laver omelet af sine guldæg	The municipality makes an omelet from its golden eggs	Helsingør Dagblad	11 January 2025
20. Der bliver flere ensomme danskere. Kan man gøre mere politisk?	There are more lonely Danes. Can more be done politically?	Kristeligt Dagblad	25 January 2025
21. Det levede liv er ikke dyrt, det er først dyrt, når I sparer det væk	Life lived is not expensive, it is only expensive when you make savings	Frederiksborg Amts Avis	27 March 2025
22. Den menneskelige kontakt er en absolut nødvendighed for at undgå ensomhed	Human contact is an absolute necessity to avoid loneliness	Dagbladet Køge	27 March 2025
23. Debat. Jeg er ikke sur - jeg er bekymret for folkesundheden i Guldborgsund	Debate. I'm not mad - I'm worried about public health in Guldborgsund	Lolland-Falsters Folketidende	12 April 2025
24. De startede ungdomsoprøret. Nu er de i gang med et buldrende ældreoprør	They started the youth revolt. Now they are starting a rumbling elder revolt.	Politiken	12 April 2025
25. Tid med mig selv	Time for myself	Weekendavisen	16 April 2025
26. Sundhedstjenestens direktør foreslår besøgsvenner til ensomme ældre	The director of the health service suggests visiting friends for lonely older adults	Flensborg Avis	5 May 2025
27. Kommunen modtager millioner til bekæmpelse af ensomhed.	The municipality receives millions to combat loneliness.	Greve-Solrød	9 May 2025
28. Råd vil have undersøgt omfanget af ældre med misbrug	Council wants to investigate the extent of older adults with drug abuse	JydskeVestkysten Kolding	19 June 2025
29. Det vilde store partier i Helsingør bruge penge på næste år	What major parties in Helsingør would spend money on next year	Helsingør Dagblad	27 August 2025
30. »Det er vigtigt, at du ikke får en slap muskeldragt«	"It's important that you don't get a loose muscle suit"	Politiken	27 September 2025
31. Ensomhed blandt ældre skal forebygges bedre - Sønderborg viser vejen	Loneliness among older adults to be better prevented - Sønderborg shows the way	JydskeVestkysten	30 September 2025
32. LA:Pårørende skal stå for personlig pleje	LA: Relatives must be responsible for personal care	Lolland-Falsters Folketidende	2 October 2025

Article Title (Original in Danish)	English Translation of Article Title	Newspaper	Publication Date
33. Værdighed. Socialdemokratiet svigter: Livskvalitet til ældre er ikke en luksus, men en nødvendighed!	Dignity. The Social Democrats fail: Quality of life of older adults is not a luxury, but a necessity!	Randers Amtsavis	4 October 2025
34. Den sværeste flytning: Plejehjemmet som sidste hjem	The hardest move: The nursing home as the last home	Vejle Amts Folkeblad	23 October 2025
35. Ældre. Fakta om borgere over 67 år i Helsingør Kommune	Older adults. Facts about citizens over 67 years of age in Helsingør Municipality	Helsingør Dagblad	29 October 2025
36. Regeringen vil sikre flere boligfællesskaber til ensomme ældre	The government will ensure more housing communities for lonely older adults	Berlingske	7 November 2025
37. Flere ensomme i mindretallet: Sådan kan vi bekæmpe det	More lonely people in minorities: How we can fight it	Flensborg Avis	7 November 2025
38. Stadig nul kroner til søndagscaféer	Still zero crones to Sunday café	Herning Folkeblad	10 November 2025
39. LA: -Ny politisk ledelse skal løfte Roskildes ældrepleje	LA: -New political leadership must improve Roskilde's eldercare	Sjællandske Nyheder	10 November 2025
40. Byrådskandidat: Nu kan der ikke skæres mere	City Council Candidate: Nothing left to cut back on	Helsingør Dagblad	17 November 2025
41. DF-veteran tilbage i Helsingør	DF veteran back in Helsingør	Helsingør Dagblad	22 November 2025
42. Dorte og Claus holdt jul med en fremmed: Rekordmange er ensomme i julen	Dorte and Claus spent Christmas with a stranger: A record number of people are lonely at Christmas	Kristeligt Dagblad	29 November 2025
43. Er julen svær, så lad nytåret blive bedre	If Christmas is hard, let the New Year be better	Frederiksborg Amts Avis	16 December 2025
44. Julens glædelige budskab	The joyful message of Christmas	Midtjyllands Avis	22 December 2025
45. Du kan tro, hvad fanden du vil – men nogen står altid klar til at gribe dig	You can believe whatever the hell you want – but someone is always ready to grab you	Århus Stiftstidende	27 December 2025
**Discourse of housing and the importance of the physical environment for reducing loneliness**			
1. Ekspert: Flere seniorbofællesskaber er på vej	Expert: More senior housing communities are on the way	Århus Stiftstidende	12 April 2017
2. Bofællesskaber tiltrækker ældre	Residential communities attract older adults	Fyns Amts Avis	18 April 2017
3. Ensomhed dræber: Alligevel vil ældre helst bo alene	Loneliness kills: Yet older people prefer to live alone	Ekstra Bladet dk	18 September 2017
**Discourse of lived experiences of loneliness in later life**			
1. Skulle jeg bare sidde her og gå fuldstændig i stå	Should I just sit here and come to a standstill	Dagbladet Holstebro-Struer	19 January 2017
2. Grethe har ingen at dele sorgen med: »Med fjernsynet tændt er der ligesom lidt selskab«	Grethe has no one to share her grief with: "With the television on, there's a little company at least."	Jyllands-Posten	13 July 2017
